# Genome sequence generated by hybrid Nanopore-Illumina assembly of an extensively drug-resistant *Pseudomonas aeruginosa* strain from a keratitis outbreak

**DOI:** 10.1128/mra.01188-23

**Published:** 2024-01-24

**Authors:** Rachel C. Calvario, Robert M. Q. Shanks

**Affiliations:** 1 Department of Ophthalmology, Charles T. Campbell Laboratory of Ophthalmic Microbiology, University of Pittsburgh School of Medicine, Pittsburgh, Pennsylvania, USA; The University of Arizona, Tucson, Arizona, USA

**Keywords:** *Pseudomonas aeruginosa*, keratitis, outbreak, infection, genome, antibiotic resistance, extensively drug resistant

## Abstract

Here, we report the genome sequence of an extensively drug-resistant *Pseudomonas aeruginosa* associated with a keratitis outbreak. This isolate was from the Center for Disease Control and Prevention and originates from a cornea. This sequence will facilitate studies of extensively drug resistant bacteria.

## ANNOUNCEMENT

Early in the year 2023, the CDC announced an outbreak of an extensively drug-resistant *P. aeruginosa* strain causing many infections, notably keratitis associated with contaminated artificial tears (1, 2). Infection by this strain has led to blindness and death in several cases throughout the United States (1, 2). It carries genes for Verona integron-mediated metallo-β-lactamase and the Guiana extended-spectrum β-lactamase which has not been formerly seen in the United States. This bacterium is highly resistant to standard of care antibiotics for *P. aeruginosa* keratitis (ciprofloxacin and tobramycin) with minimum inhibitory concentrations above 32 µg/mL (1, 3). Here, we report the genome of an isolate from this outbreak using whole genome sequencing (WGS) made using Illumina and Nanopore and assembled with Unicycler software.

The isolate was provided by the CDC and was collected from a patient’s cornea in 2022. MIC data are reported on the CDC’s website that describes antibiotic susceptibility for this isolate. Here, we investigate isolate “AR-1270,” [also noted as CDC1270 (1, 3)]. The CDC assigned BioSample ID SAMN33435243 to this isolate. The sample was streaked onto a Blood Agar Plate and incubated overnight at 37˚C. A single colony from this plate was subsequently grown in 5 mL of lysogeny broth on a rotary shaker at 30˚C overnight. Genomic DNA was extracted using the Qiagen Puregene Cell Core Kit.

Sequencing was performed by SeqCenter, LLC. A tagmentation- and PCR-based Illumina DNA Prep kit with custom IDT 10 bp unique dual indices (UDI) libraries was used to prepare libraries. This kit utilized Illumina NovaSeq 6000 sequencer and bcl-convert1 (v4.1.5) for adapter trimming and quality control. For long reads, PCR-free Oxford Nanopore Technologies (ONT) Ligation Sequencing Kit (SQK-NBD114.24) with the NEBNext Companion Module (E7180L) was used to make sample libraries according to the manufacturer’s specifications. The DNA was not fragmented or size-selected further. A MinION-Mk1B sequencer or a GridION sequencer using R10.4.1 flow cells was used for sequencing. Run design utilized the 400 bps sequencing mode with a minimum read length of 200 bp. Guppy1 (v6.4.6) was used for super-accurate base calling (SUP), demultiplexing, and adapter removal (dna_r10.4.1_e8.2_400bps_modbases_5mc_cg_sup.cfg).

Unicycler (v0.4.8) was used to assemble the genome from both sequencing results. Pilon (v1.23) and Racon (v1.4.20) were used for polishing, and trim_galore (v0.6.5dev) was used for preprocessing. Other tools that were used are Bandage (v0.8.1), minimap2 (2.17-r974-dirty), and samtools (v1.17 [using htslib 1.17]). Default parameters were used. The genome was then annotated by the NCBI Prokaryotic Genome Annotation Pipeline (PGAP, v.6.3) (4).

One contig was generated for this genome, being 7,082,821 bp in length. The genome consists of 6,720 protein coding sequences. Of which, 1,509 were hypothetical and 5,211 were given functional assignments. It had intact *ladS* (5) and *lasR* (6) regulatory genes which are sometimes mutated in clinical isolates.

PATRIC’s Comprehensive Genome Analysis found 70 antibiotic resistance genes using The Comprehensive Antibiotic Resistance Database. Given the broad antibiotic resistance capabilities of this organism, further analysis was done using CARD (v.3.8.8). There are 20 perfect hits of resistance genes that match 100% identity to reference sequences, and 55 strict hits, with sequences matching from 32.33% to 100% identity to the reference sequence (Table 1).

**TABLE 1:** Statistics regarding the sequencing, assembly, annotation, and characterization of *Pseudomonas aeruginosa* strain AR-1270

Illumina sequencing	Base pairs generated	4.73 × 10^8^
	Paired ends generated	2 × 151 bp
Oxford nanopore sequencing	Reads	72,637
	Total base pairs	346,460,949
Assembly	Number of contigs	1
	Length	7,082,821
	Long read coverage	48
	Short read coverage	71
	GC content	65.87%
	*N*_50_	7,082,821
Annotation	CDS	6,720
		Hypothetical: 1,509
		Assigned function: 5,211
	Transfer RNA genes	64
	Repeat regions	63
	Ribosomal RNA genes	12
Antibiotic resistance genes	CARD (7)	Total: 70
		Perfect hits: 20
		Strict hits: 55
	NDARO (8)	25
	PATRIC (9)	105
Other analyses	MLST type (10)	1203
	Prophage regions (11)	Regions found: 5
		Intact: 1

## Data Availability

This genome project has been submitted to GenBank under the accession number CP137556. The version described in this paper is the first using Nanopore-Illumina hybrid sequencing (CP137556.1) but has previously been sequenced by the CDC. The genome and information is available by Genome accession number: CP137556, BioProject: PRJNA1031647, and BioSample: SAMN37951956.
